# Digital Image Correlation and Ultrasonic Lamb Waves for the Detection and Prediction of Crack-Type Damage in Fiber-Reinforced Polymer Composite Laminates

**DOI:** 10.3390/polym16141980

**Published:** 2024-07-11

**Authors:** Elena Jasiūnienė, Tomas Vaitkūnas, Justina Šeštokė, Paulius Griškevičius

**Affiliations:** 1 Prof. K. Barsauskas Ultrasound Research Institute, Kaunas University of Technology, LT-51423 Kaunas, Lithuania; justina.sestoke@ktu.lt; 2 Department of Electronics Engineering, Kaunas University of Technology, Studentu Str. 50, LT-51368 Kaunas, Lithuania; 3 Department of Mechanical Engineering, Kaunas University of Technology, Studentu Str. 56, LT-51424 Kaunas, Lithuania; tomas.vaitkunas@ktu.lt (T.V.); paulius.griskevicius@ktu.lt (P.G.)

**Keywords:** crack damage identification, fiber reinforced polymer composite laminate, finite element modeling, finite element updating, digital image correlation, ultrasonic lamb waves

## Abstract

The possibility of using the Digital Image Correlation (DIC) technique, along with Lamb wave analysis, was investigated in this study for damage detection and characterization of polymer carbon fiber (CFRP) composites with the help of numerical modeling. The finite element model (FEM) of the composite specimen with artificial damage was developed in ANSYS and validated by the results of full-field DIC strain measurements. A quantitative analysis of the damage detection capabilities of DIC structure surface strain measurements in the context of different defect sizes, depths, and orientation angles relative to the loading direction was conducted. For Lamb wave analysis, a 2D spatial-temporal spectrum analysis and FEM using ABAQUS software were conducted to investigate the interaction of Lamb waves with the different defects. It was demonstrated that the FEM updating procedure could be used to characterize damage shape and size from the composite structure surface strain field from DIC. DIC defect detection capabilities for different loadings are demonstrated for the CFRP composite. For the identification of any composite defect, its characterization, and possible further monitoring, a methodology based on initial Lamb wave analysis followed by DIC testing is proposed.

## 1. Introduction

Fiber reinforced polymer composites are attractive in many industries due to their superior strength, fatigue resistance, and low-mass material properties, although defects can significantly reduce these structural properties. It is possible for composite defects to form during the manufacturing process or for damages during the exploitation period. Consequently, the presence of defects/damages in composite structures is not always avoidable. Even barely visible impact damage, which is difficult to identify, can grow, leading to catastrophic failure of the material [1]. The identification and monitoring of composite defects/damages (further, both are called defects) are important tasks for structure health monitoring (SHM) [2]. Multiple different methods have been developed and employed for the testing of composite structures. These include, but are not limited to, acoustic emission [3], ultrasound testing [4], X-ray computed tomography [5], infrared thermography [6], etc. Although the aforementioned methods are accurate, they have some limitations. These include contact with the tested structure, special testing conditions for X-ray scans, and heating for thermography, which prevents them from being widely adopted in the SHM of composites.

Non-contact digital image correlation (DIC) as a full-field, novel optical measurement technique was developed in 1980 [7]. Test object surface points are tracked by optical cameras, and numerical calculations are used to determine object surface displacements and strains. A two-camera setup can even capture out-of-plane surface displacements (3D DIC [8]). DIC, as a simple and attractive method, has already been used to identify the material properties of different materials [9], track crack growth [10], and determine the failure of materials [11]. Studies of defect identification in composite structures have already been carried out using DIC measurements of the composite surface at different loads [12,13,14,15]. Defects can be identified by unevenness in displacements or strain fields. For example, Ambu [16] assessed the progression of damage in graphite/PEEK and graphite/epoxy laminates using strain fields from DIC measurements, and the inhomogeneities in strain maps from DIC were used to identify delamination. Gonabadi et al. [17] used DIC to track fatigue damage in glass fiber composites. Defects are easier to identify in the structure surface strain field because surface displacement fields require a measurement resolution of several micrometers to identify defects, as demonstrated in [18]. However, the noise in the strain reduces the accuracy of the DIC strain [19], so the unevenness in the DIC strain field cannot be distinguished between the small defect and the measurement noise. The depth of damage in the structure and the type of loading have a significant influence on the ability to detect damage from the DIC surface strain field. For example, under tensile loading, fiber cracks are easier to detect [13,14] in contrast to compression loading, where local buckling causes light reflection and greatly increases measurement noise [14]. The DIC surface displacement or strain fields measured during bending tests can be used to identify other types of composite damage, such as delamination or debonding [12]. Nevertheless, damage that is located far from the surface of the structure may not even be visible on the surface strain or displacement field. In such cases, additional testing methods may be required.

The use of ultrasonic guided Lamb waves for the inspection of various structures is also becoming increasingly popular. Lamb waves have been proven to be effective in detecting various types of damage in structures [20,21,22]. However, the suitability of guided Lamb waves for the inspection of a particular structure is dependent on a number of factors, including, but not limited to, the material from which the structure is made, the thickness of the structure, etc. Inspection of anisotropic materials such as fiber-reinforced composites presents even more challenges due to the propagation of Lamb waves at different speeds in different directions. Furthermore, it is common that multiple modes exist that have dispersive properties, making the identification of defects challenging [22]. The presence of multiple Lamb wave modes, which are reflected from structural boundaries, can result in the masking of indications of the defects [22]. The dominant displacement of the particles varies depending on the mode of propagation of the Lamb waves. Consequently, for the detection of delaminations, anti-symmetric modes with predominant out-of-plane displacements are usually employed [23], whereas for the detection of cracks, symmetric modes with a dominant in-plane displacement are more effective [24]. The same order of antisymmetric modes has a shorter wavelength than that of symmetric modes, which leads to enhanced sensitivity in terms of smaller defect sizes [24]. Therefore, Lamb waves are frequently used for the identification of delamination-type defects in composite structures [25,26,27,28].

The ability of DIC and Lamb waves to detect damages is dependent upon several damage parameters, including the structure’s size, loading type, relative direction to the damage orientation, and depth within the structure. Studies analyzing DIC damage detection capabilities in relation to damage parameters have not yet been conducted. In addition, there has been considerably less research into the use of Lamb waves for crack detection, particularly in composite structures [29,30]. The aim of this study is to combine two different damage identification techniques, namely DIC and Lamb waves, and to develop a methodology for the identification and characterization of damage. The Lamb waves technique is going to be applied for the damage identification, and the DIC system for the damage characterization [29,30].

## 2. Materials and Methods

### 2.1. Test Specimens

**Figure 1:**
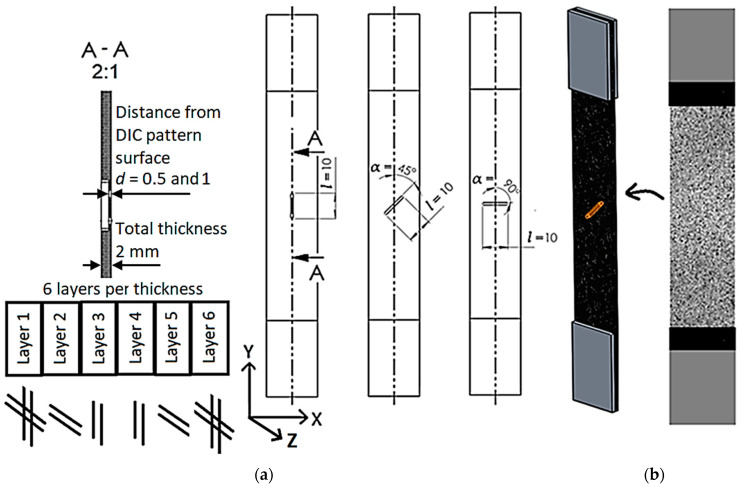
CFRP specimen according to standard ASTM D3039: (**a**)—artificial defects (milled notches); (**b**)—pattern for DIC measurements.

Standard carbon fiber reinforced polymer (CFRP) composite test specimens with different types of crack damage were prepared for this study. Press molded OKE carbon fiber plates (350 × 150 × 2 mm) made from high-tenacity carbon fiber prepregs with transparent epoxy resin were used. External plies are made of weave twill 3-K carbon prepreg 200 g/m^2^, and core plies are made of 0/90 carbon unidirectional prepregs. CFRP plate consists of a total of 6 layers with the fiber orientation angles [(0/90)°/90°/0°]s. For the DIC strain analysis, the specimens were cut from a CFRP plate with a layup of [(0/90)°/90°/0°]s on a circular table saw at an angle of 0 degrees, as shown in Figure 1a, and their dimensions were taken according to ASTM D3039 [31].

While the specimen width of 25 mm was determined according to the ASTM D3039 standard, there are no direct requirements for the specimen length. Therefore, a specimen length of 150 mm was selected as the CFRP composite plate dimension. Aluminum tabs of 1.5 mm thickness were bonded to the specimen’s ends to prevent specimen failure in the grip of the machine. Artificial defects—fiber cracks—were formed by milling notches of 1.5 mm and 1 mm depth corresponding to *d* = 0.5 and 1 mm distances from the surface with DIC pattern, with a length of 10 mm and orientation angles to the tensile direction of 0, 45, and 90°, as shown in Figure 1a. A specimen with a speckle pattern for DIC measurements is shown in Figure 1b.

### 2.2. Calculation of Guided Wave Dispersion Curves Using the Semi-Analytical Finite Element (SAFE) Method

This section presents the theoretical investigation using semi-analytical finite element (SAFE) calculations focused on the propagation of guided wave modes in an OKE CFRP plate of dimensions 350 × 150 × 2 mm. The aim of this theoretical investigation was to determine the best excitation frequency, which would enable us to efficiently excite and receive the required Lamb wave mode to locate and identify defects in a composite specimen using a single-element ultrasonic transducer. First of all, theoretical calculations using the semi-analytical finite element (SAFE) method were performed. The SAFE method can be used for layered orthotropic material. The orientation of the specimen for SAFE calculations is the same as shown in Figure 1 for DIC strain analysis. The position of the specimen in the coordinate system is as follows: the direction of wave propagation is in the *Y* direction; the width of the sample is 150 mm (corresponding to the *X* direction); and the thickness of the sample is 2 mm (corresponding to the *Z* direction). The definition of the material properties of the CFRP specimen model is based on the rule of mixture calculations, according to the elastic modulus of fiber *E_f_* = 230 GPa and the matrix *E_m_* = 2.45 GPa, Poisson’s ratios of fiber *ν_f_* = 0.18, and matrix *ν_m_* = 0.4, as well as the fiber volume fracture *V_f_* = 0.6 [15]. The material stiffness matrix is defined as follows:

(1) $$C=\left[\begin{array}{cccccc} 74.8 & 2.66 & 0.36 & 0 & 0 & 0\\ 2.66 & 74.8 & 0.36 & 0 & 0 & 0\\ 0.36 & 0.36 & 9.82 & 0 & 0 & 0\\ 0 & 0 & 0 & 3.04 & 0 & 0\\ 0 & 0 & 0 & 0 & 3.04 & 0\\ 0 & 0 & 0 & 0 & 0 & 3.39 \end{array}\right]\;\mathrm{GPa}$$

**Figure 2:**
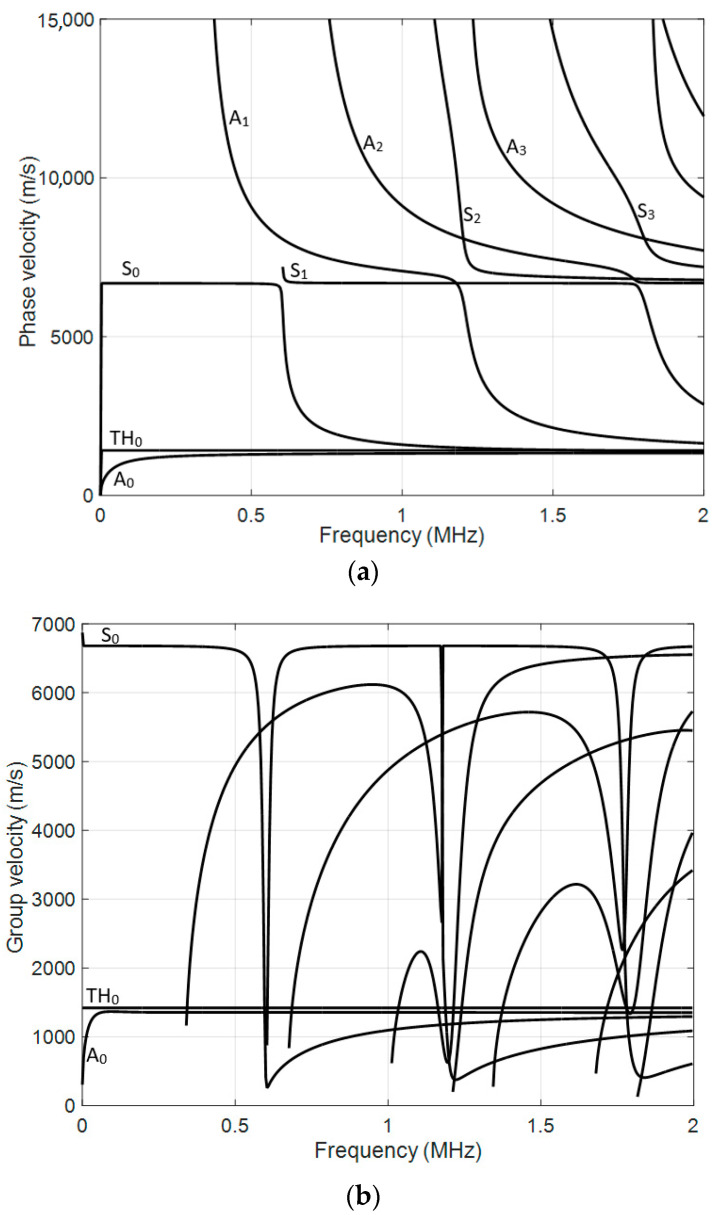
Calculated dispersion curves of the CFRP 2 mm sample: (**a**) phase velocities; (**b**) group velocities.

The simulated dispersion curves of different guided Lamb modes for the selected specimen are presented in Figure 2a—the phase velocity, and Figure 2b—the group velocity. In dispersive media, such as composites, the phase velocity and group velocity of ultrasonic waves are functions of frequency. As the frequency changes, so does the relationship between phase and group velocity. In addition, more modes are excited at higher frequencies. Therefore, prior to any theoretical or experimental investigation, the phase and group velocities of the frequency-dependent modes of guided Lamb waves in the existing structure should first be determined. By knowing the sample phase velocities from the dispersion curves, it is possible to determine at which frequency the Lamb modes should be excited. For example, in this case study, we are interested in the antisymmetric mode *A*_0_ and the symmetric mode *S*_0_. By selecting suitable ultrasonic transducers, we can identify cracks and defects in the sample. It can be seen from the dispersion curves that for this particular sample at the frequency of 500 kHz, *A*_0_, *TH*_0_, *S*_0_, and *A*_1_ modes can be excited.

### 2.3. Digital Image Correlation

**Figure 3:**
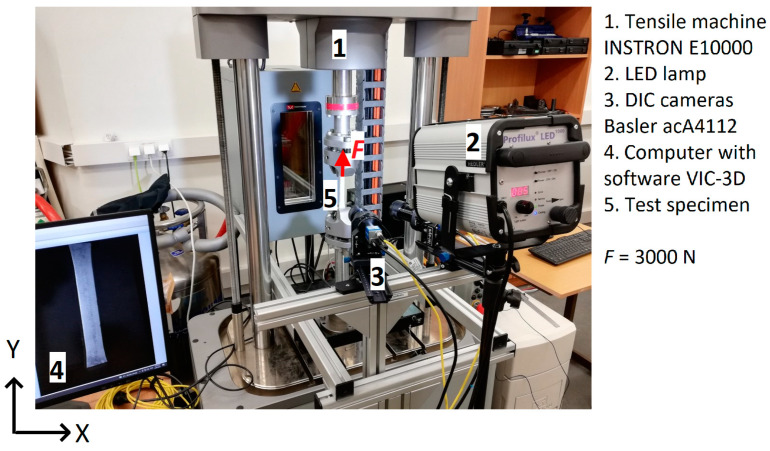
DIC strain measurement setup for the CFRP specimen.

The speckle pattern on the surface of the specimen, necessary for the DIC measurements, was created by painting. Black paint was sprayed with an airbrush, resulting in black dots of size 0.05 mm on the previously white paint-coated specimen surface. The specimen was loaded with static tensile force in the *Y* direction using the universal testing machine (ElectroPuls E10000, Instron, Norwood, MA, USA) (Figure 3). Images of the deformed and undeformed specimen surfaces were captured with two DIC cameras acA4112-20um (Basler AG, Ahrensburg, Germany). To avoid defect growth or specimen failure, the sample was loaded up to 3000 N (7.5% specimen failure load). Lightening was created with a 1000W maximum power LED lamp (Hedler Profilux LED 1000). The experimental setup photo is shown in Figure 3.

A 3 mm (9 × 9 points) DIC calibration plate was used to calibrate the DIC system. 26 calibration images were taken, giving a calibration score of 0.047. DIC images were post-processed by using a DIC step size of 11 px (0.09 mm), a subset size of 57 px (0.46 mm), and an 11% strain filter in the VIC-3D-9 software, resulting in a measurement uncertainty interval of 0.02 px.

### 2.4. Numerical Modeling of the CFRP Specimen Surface Strains

**Figure 4:**
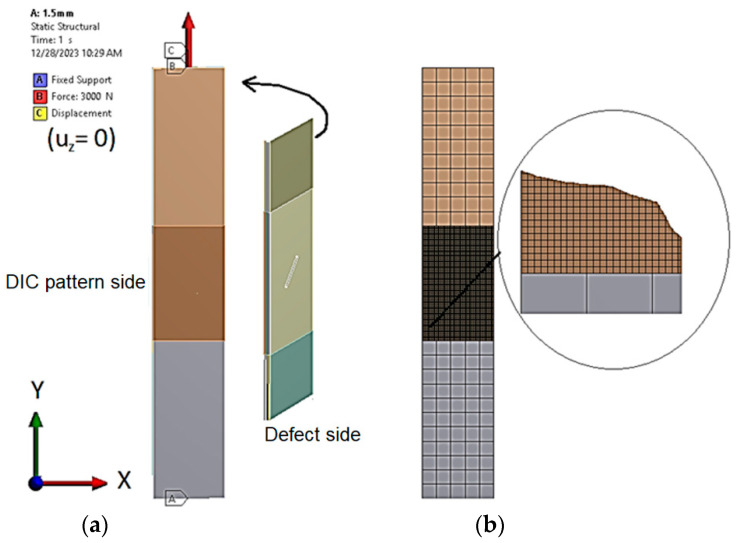
Numerical modeling of a CFRP specimen with a defect depth *d* = 0.5 mm from the surface (notch depth 1.5 mm): (**a**)—boundary conditions; (**b**)—FE mesh.

The finite element (FE) model of a CFRP specimen with a defect, located at *d* = 0.5 mm from the surface, was created in ANSYS Workbench 19.1 software. The model structure and boundary conditions are shown in Figure 4.

The model (Figure 4a) of the CFRP specimen consists of 6 composite layers per thickness, resulting in the thickness of each layer being 0.333 mm. FEM as a single-layer SHELL element formulation is only valid for layer-wise defect depths; therefore, it is not possible to model any value of defect depth. To address this issue, the CFRP specimen was modeled using SOLID elements, and each composite layer was defined as a separate body with anisotropic material (Figure 4). The CFRP specimen model boundary conditions replicate those in the experiment: the bottom of the specimen is fixed, while the top of the specimen is fixed in the *Z* direction and loaded with a tensile force of 3000 N in the *Y* direction (Figure 4). Because the defect dimensions (notch width of 1 mm) are relatively small compared to the specimen dimensions, a fine FE mesh is required in the defect zone, whereas elsewhere a mesh of square elements of 5 mm size is sufficient. Split lines were used in ANSYS to divide the virtual CFRP specimen and include a defect zone with a 0.5 mm square mesh. Contact type “Bonded” was selected to connect all bodies in the model.

## 3. Results and Discussion

### 3.1. FEM Simulation of Lamb Wave Propagation

**Figure 5:**
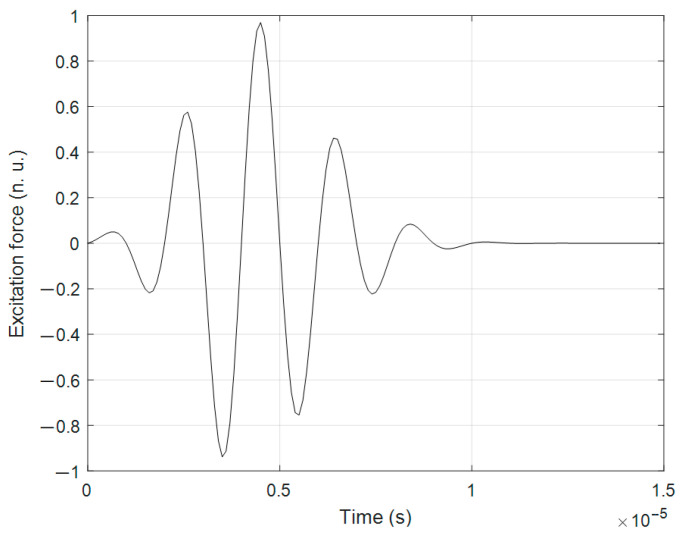
The excitation signal.

**Figure 6:**
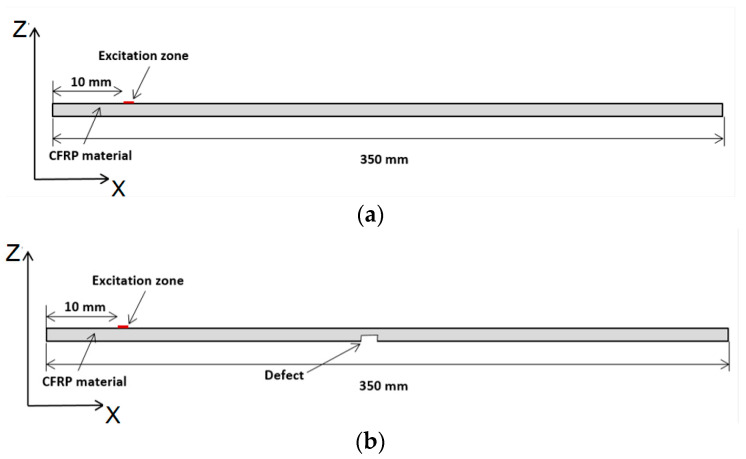
The simulation diagram by the FEM method: (**a**) the CFRP specimen without defect; (**b**) the specimen with defect.

Another calculation method, a FEM simulation, was focused on the modeling of propagation of guided Lamb wave modes in the sample—a CFRP plate of dimensions 350 × 150 × 2 mm. The aim of this theoretical calculation was to simulate excited Lamb wave mode interactions with different defects. The ABAQUS software 7.00 package (Dassault Systems, Johnston, RI, USA) was employed for the simulation of the propagation of the guided wave modes in the CFRP specimen. The ABAQUS program was selected for its high level of detail, flexibility to configure specimens, and accurate mesh control. By applying the 2D calculation method, Lamb wave modes in the composite material were excited. The excitation frequency was set at *f* = 500 kHz, with a burst of three periods. The excitation signal is presented in Figure 5. The direction of wave propagation in this model is in the *X* direction (Figure 6), the length of the sample in this direction is 350 mm, and the thickness of the sample is 2 mm (corresponding to the *Z* direction).

The simulation diagram in Figure 6a illustrates the simulated setup. The excitation zone was at the distance *X* = 10 mm from the left edge of the CFRP specimen and was equal to 2.5 mm.

**Figure 7:**
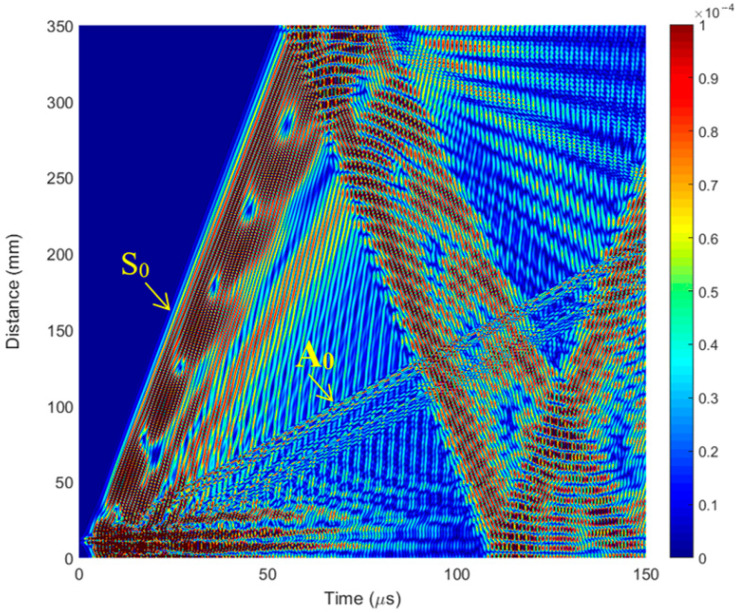
The simulated B-scan without defects of the CFRP specimen.

The aim of this theoretical investigation using the FEM method was to simulate Lamb wave propagation and interaction with structure in the case of a perfect sample and a sample with defects and detect defects located at different depths in a CFRP composite specimen using a single-element ultrasonic transducer. In the 2D model (Figure 6b), the defect is located in the middle of the specimen, and the l, and a defect width of 1 mm was chosen with 3 different distances from the surface: *d* = 1.5 mm, 1.0 mm, and 0.5 mm (notch depths of 0.5, 1, and 1.5 mm). (Figure 6b). The simulated B-scan without defects is presented in Figure 7. The B-scan is a two-dimensional cross-sectional view of an object under test. The B-scan was acquired over the full length of the specimen, i.e., 350 mm in the X-direction (direction of wave propagation).

From the simulated B-scan, it can be determined which mode (or modes) are excited by a transducer. It could be performed by calculating the wave velocity using the method where the phase velocities are determined from the distances ∆*l_gr_* and ∆*l_ph_* (*Y* coordinate axis) traveled by the corresponding specific phase point during the corresponding time intervals ∆*t_gr_* and ∆*t_ph_* (*X* coordinate axis):

(2) $$c_{ph}=\frac{∆l_{ph}}{∆t_{ph}},$$

**Figure 8:**
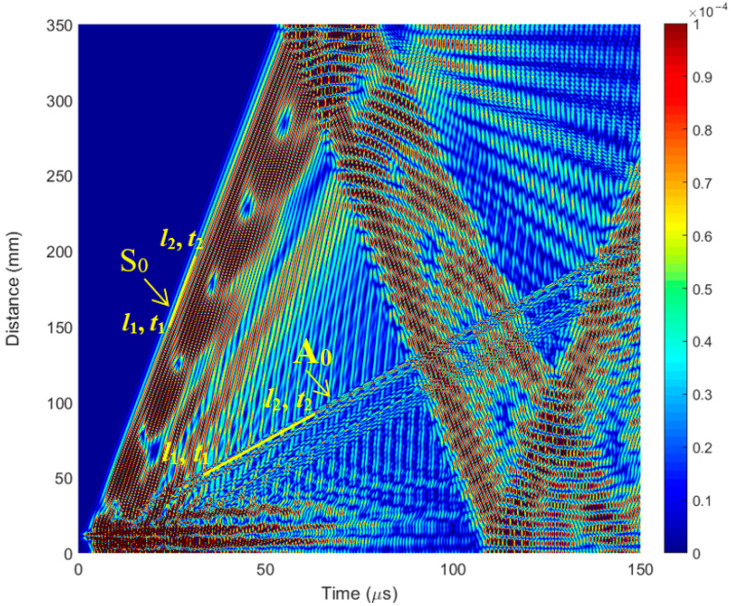
The simulated B-scan of the CFRP showing propagating modes.

The phase velocity value calculated from the numerical simulation results for the first wave packet is *c_ph_* = 5856 m/s (Figure 8). In comparison, the phase velocity of the *S*_0_ mode estimated by the SAFE method is *c_ph_* = 5829 m/s (Figure 2a). Therefore, it can be concluded that the first wave packet is in *S*_0_ mode. The phase velocity value calculated for another, slower wave mode from the numerical simulation results is *c_ph_* = 1369 m/s (Figure 8). In comparison, the phase velocity of the *A*_0_ mode estimated by the SAFE method is *c_ph_* = 1421 m/s (Figure 2a). It can be concluded that there is an *A*_0_ mode propagating with a phase velocity of *c_ph_* = 1369 m/s.

**Figure 9:**
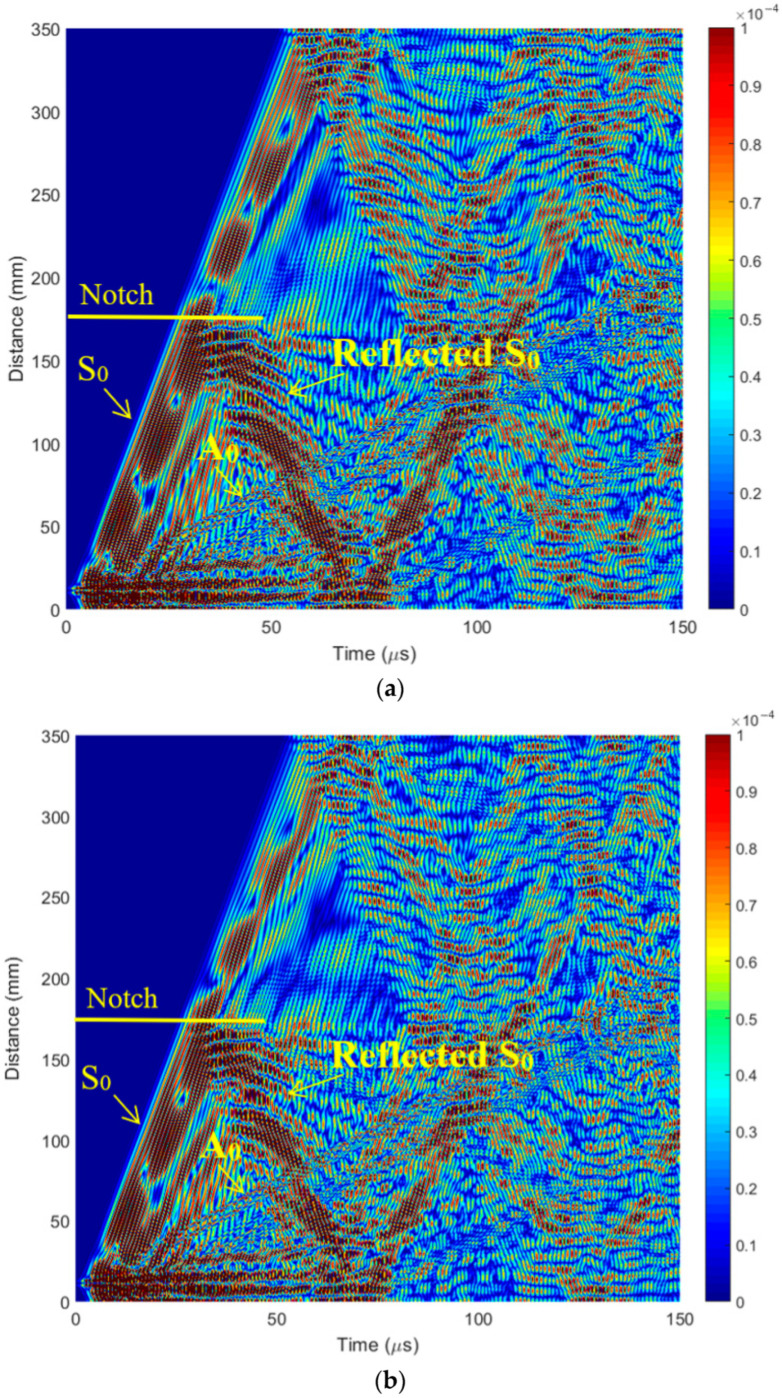

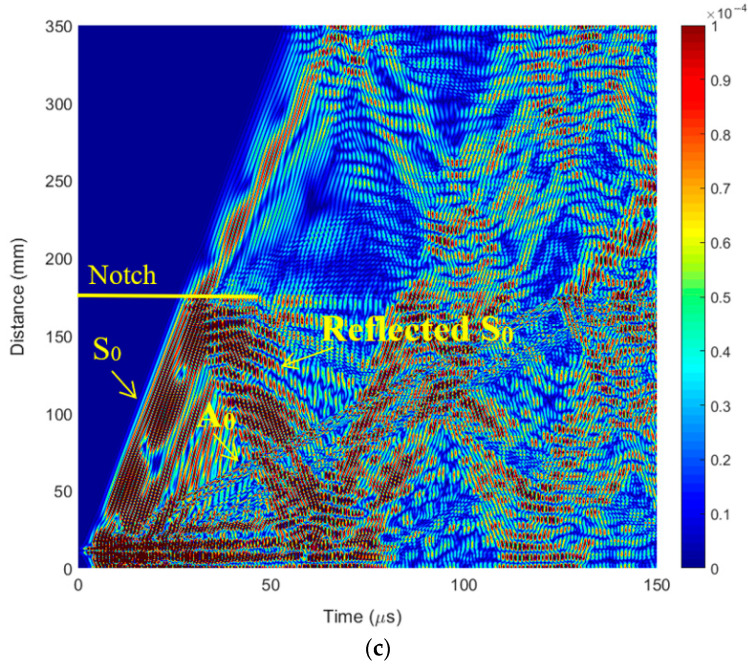
The simulated B-scan with different defect distances from the surface: (**a**) *d* = 1.5 mm; (**b**) *d* = 1.0 mm; (**c**) *d* = 0.5 mm.

The simulated B-scans with different defect depths are presented in Figure 9. In the model (Figure 6b), the defect is located in the middle of the specimen, and the defect width is 1 mm. A notch was formed on the lower surface of the specimen in the middle of the specimen, and B-scan data were acquired on the upper layer of the specimen by recording the ultrasonic echoes received. From the simulation results (Figure 9), it is clearly visible that the defect is located at the center line of the specimen (175 mm) (Figure 9a). The reflected S_0_ Lamb wave indicates the presence of a defect for all defect sizes (depths).

### 3.2. Digital Image Correlation Results

#### 3.2.1. Finite Element Model Validation and Defect Detection

**Figure 10:**
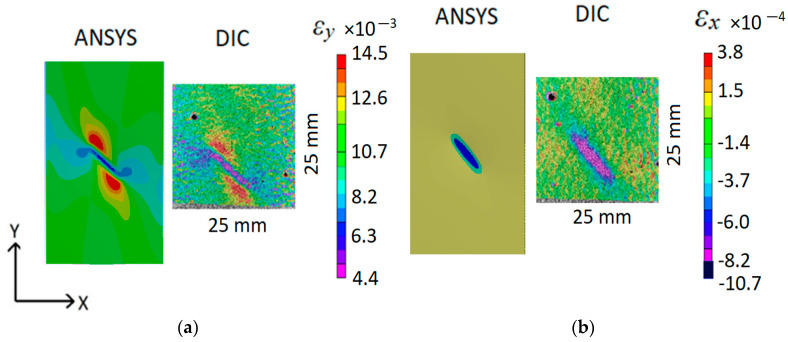
Experimental (DIC) and ANSYS simulated CFRP specimen surface strain fields with the *d* = 0.5 mm, *α* = 45°, *l* = 10 mm defect: (**a**)—tensile direction *Y*; (**b**)—transverse direction *X*.

A comparison of experimental DIC and ANSYS simulated strain fields for a static tensile loading of 3000 N force is shown in Figure 10.

The simulated ANSYS and experimental DIC strain fields show agreement in both color and shape, confirming the validity of the CFRP specimen’s FEM. However, there are visible strain differences at the center of the defect, but they do not have an impact on further analysis. In the case of longitudinal strain, ANSYS simulated *ε_y_min_* = 8.2 × 10^−3^, whereas the minimum DIC tensile strain for the same case was *ε_y_min_* = 4.4 × 10^−3^. Similarly, for transverse strains, the minimum value of *ε_x_min_* = −10.7 × 10^−3^ for ANSYS, while the minimum value for DIC was *ε_x_min_* = −8.2 × 10^−3^. High strain gradients at the defect center location are primarily responsible for the differences, and to accurately capture those strain gradients, the FE mesh needs to be even finer. It should also be noted that the strain accuracy of DIC is approximately 10% [32], thus, strain differences of this magnitude between DIC and FEM are possible.

**Figure 11:**
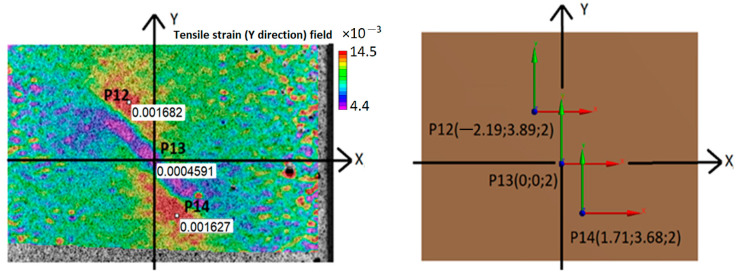
Defect depth in CFRP specimen calculation with DIC and FEMU: selected points on the specimen surface for comparison of strain values in the *Y* direction.

Once the composite defect is visible in the DIC composite surface strain fields (Figure 10), the DIC strain values can be used to find unknown defect parameters, such as distance from the structure surface, location, and size. This is determined in ANSYS, and the depth of the notch is selected as a parameter (P1 in ANSYS, Figure 11) to be found by finite element model updating (FEMU). The location and shape of the defect for this particular loading can be determined directly from the DIC strain fields. The “Seek target” optimization option in ANSYS is used to find the depth of the notch in CFRP composite, ensuring that the strains at the P12-P14 points in FEM would be equal to the strains measured by DIC at the same location on the specimen surface.

The depth of the notch of 1.53 mm (distance from the surface of 0.47 mm) found by the ANSYS optimization is in very good agreement with the real depth of the defect of 1.5 mm (distance from the surface of 0.5 mm). DIC and simulated strain values are found to be in close agreement at point P12 (1.682 × 10^−3^ DIC vs. 1.705 × 10^−3^ ANSYS, Figure 11). At point P13, the FE strains were found to be approximately two times higher than the experimental ones. The specimen misalignment in the tensile machine, not the ideal defect shape or finite element mesh, could have had an effect on the strain differences at point P13. Nevertheless, the depth of the defect notch in the CFRP specimen was found with a relative error of 2%, which validates the DIC method for defect detection and FEMU for defect location and geometry definition.

**Figure 12:**
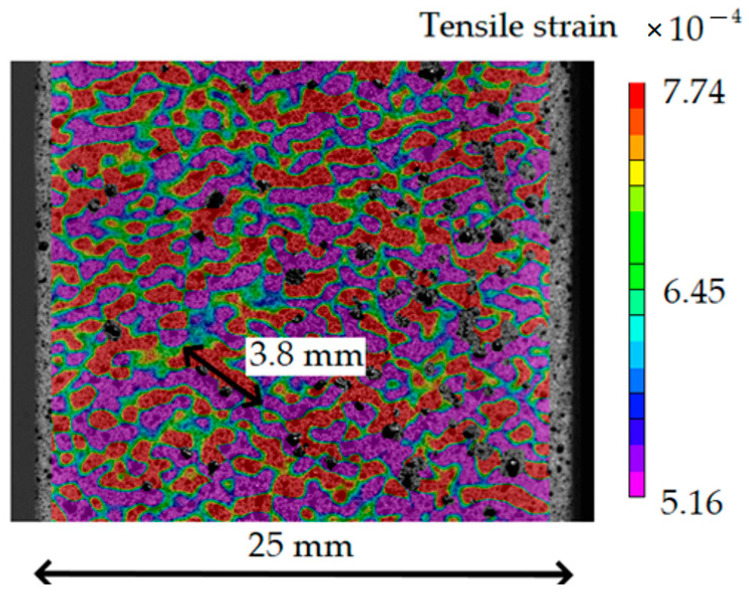
Noise induced by the roughness of the CFRP specimen surface during tensile loading.

On the other hand, not all composite defects can be found using the DIC-FEMU method. For example, defects existing deep in the structure or small defects do not have an effect on the composite strain field. In addition, defect detection by DIC is strongly related to the surface roughness of the specimen, which adds noise to the surface strain field, limiting the ability to detect defects that are small or far away from the surface. For this purpose, the noise induced by the roughness of the CFRP composite surface in the strains from the tensile strain field on the intact CFRP specimen surface was analyzed and is shown in Figure 12.

From Figure 12, it can be seen that the largest surface unevenness is 3.8 mm (15% of the specimen width). Therefore, material defects that generate strain field unevenness smaller than 3.8 mm for the current CFRP composite will be difficult to distinguish from the DIC strain field due to the surface roughness of the CFRP specimen. Theoretically, it is possible to subtract the strain field of the intact specimen from that of the specimen with defects to reduce the effect of the surface roughness and increase the DIC defect detection capabilities, but practically, it is very difficult to perform such a method on real structures (even impossible for structures with pre-existing manufacturing defects). Based on this observation, it is more effective to only test intact specimens to determine the DIC defect detection limits and select other methods for DIC nonvisible defects.

For each different composite material, its lay-up, loading, and boundary conditions, there can be an infinite number of different strain fields showing material defects. Further sub-chapters focus on DIC-validated numerical analysis of the current CFRP composite for the most common loading types: tensile loading with the same boundary conditions as in tensile machines and cantilever beam bending, to analyze defect detection limits based on defect size, distance from the structure surface, and orientation to loading.

#### 3.2.2. DIC Defect Detection Analysis for Tensile Loading

The CFRP specimen ANSYS model with the same boundary conditions, as shown in Figure 4, was used for tensile loading analysis. The maximum to average tensile strain ratio *r* = *ε_max_*/*ε_avg_* in the area of 10 mm × 10 mm around the defect was selected as the defect characterization criteria. The strain ratios for different combinations of defect parameters (angles *α =* 0°, 30°, 45°, 60°, 90°, lengths *l* = 4 mm, 5 mm, 6 mm, 10 mm, and distances from surface *d* = 0.4 mm, 0.5 mm, 0.6 mm, 0.8 mm, 1 mm, 1.2 mm, 1.4 mm, 1.6 mm) were computed and are shown in Table 1. Based on in-house experimental observation that a notch parallel to the tensile direction is not visible in the DIC surface strain field and the ANSYS computed strain ratio for such a defect is 1.1, all defects resulting in a strain ratio of 1.1 or less in the ANSYS simulation can be treated as not visible by DIC (Table 1). Furthermore, due to the unevenness of the CFRP composite surface captured by the in-house DIC surface strain measurements on the intact specimen (Figure 12), the surface strain ratio of the intact CFRP specimen was found to be *r* = 1.2. Consequently, composite defects, which can result in strain ratios of up to 1.2, can also be treated as invisible by DIC.

**Figure 13:**
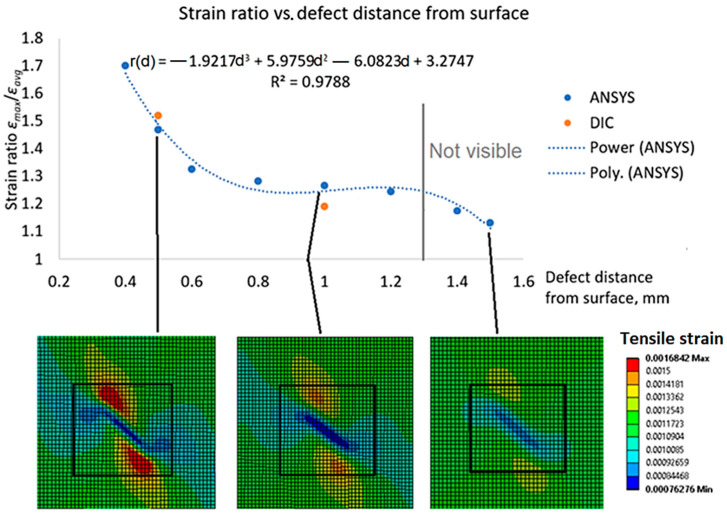
Strain ratio vs. defect distance from surface (*l* = 10 mm length, *α* = 45° angle).

The strain ratio *r* was plotted against the distance of the defect *d* from the surface (not the notch depth) in Figure 13. The relationship between strain ratio and defect distance from the surface can be approximated by several functions, including the cubic polynomial *r* = *f*(*d*) (Figure 13), which is considered to be sufficiently accurate. A sudden reduction in the strain ratio below 1.18 (Figure 13) predicts that defects 10 mm long and oriented at a 45° angle to the tensile direction located deeper than 1.3 mm (65% of the structure thickness) from the surface cannot be detected by DIC.

**Figure 14:**
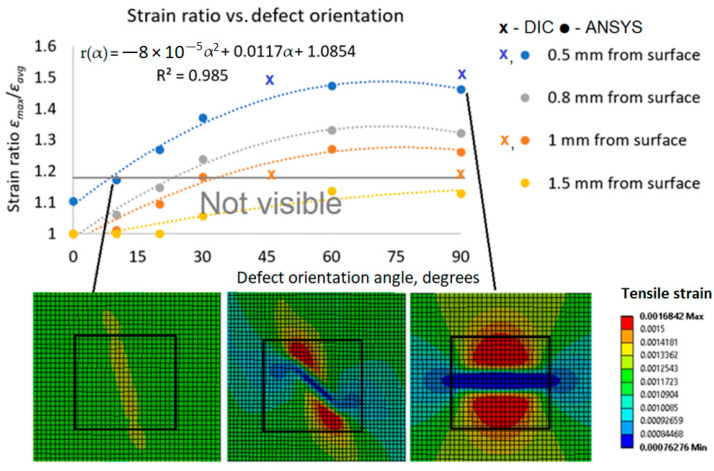
Strain ratio vs. defect orientation angle (defect length *l* = 10 mm).

The strain ratio vs. defect distance *d* relation presented in Figure 13 is valid only for a particular orientation and size of defects. It should be noted that smaller defects, oriented at different angles and located less than 1.3 mm from the surface, may not be captured by DIC. The defect orientation effect on strain ratios at different defect distances from the structure surface for a defect length of *l* = 10 mm is presented in Figure 14.

From the strain ratio to defect angle relation (Figure 14), it is possible to conclude that orientation angle has no effect on strain ratio *r* in the range of *α* = 60° ÷ 90°. The second-order polynomial approximation relating strain ratio *r* to defect orientation angle *α* is very accurate (*R*^2^ = 0.99, Figure 14). Based on the current composite analysis, for different defect distances *d* from the surface, the strain ratio proportionality is given by the following equation:

(3) $$r_{d_{1}}\left(\alpha \right)=r_{d_{2}}\left(\alpha \right)\frac{r\left(d_{1}\right)}{r\left(d_{2}\right)}$$

Thus, the minimum defect orientation angle for any defect distance from the surface *d* can be computed by knowing $r_{d_{2}}\left(\alpha \right)$ relation for particular defect depth *d*_2_ and multiplying it by the ratio $\frac{r\left(d_{1}\right)}{r\left(d_{2}\right)}$, which can be found from strain ratio *r* vs. defect distance from surface *d* relation *r*(*d*) (Figure 13).

Another parameter that is quite important is the minimum defect length, visible by DIC. The FEM results of this study indicate a linear relationship between the strain ratio *r* and the defect length *l*:

(4) $$r\left(l\right)=A\cdot l+B$$

where *A* and *B* are coefficients, which are different for each defect distance from the surface *d* and orientation angle *α* and can be found from the *r*(*l*) plot. For example, when *α* = 45° and *d* = 0.5 mm, the coefficients will be *A* = 0.0351, *B* = 1.12, and the minimum detectable defect length will be equal to 2.28 mm.

Strain ratio is also observed to be proportional to defect length and distance from the surface:

(5) $$r_{d_{1}}\left(l\right)=r_{d_{2}}\left(l\right)\frac{r\left(d_{1}\right)}{r\left(d_{2}\right)}\;$$

The average (including defects with α = 0 ÷ 90, *d* = 0.4 ÷ 1.5 mm) minimum detectable defect length of 2.5 mm (10% of the structure width) was calculated from Equations (4) and (5) for a strain ratio of *r* = 1.2. However, due to the unevenness of the CFRP specimen surface (Figure 12), the practical very minimum defect length detection capability is 3.8 mm.

#### 3.2.3. DIC Defect Detection for Bending Loading

Bending is another very common loading condition that can occur in wind turbine blades, civil structures, reservoirs, etc. The same defect parameters: distance from surface *d*, angle *α*, and length *l*, and the effect on maximum and average strain ratio *r*, were analyzed in the ANSYS model of the CFRP specimen for the bending loading case. Cantilever beam bending is considered when the bottom of the specimen is fixed and the top of the specimen is bent by a 20 N force. Based on the literature review that compression loading generates light reflection [14] and noise in DIC strains, only the case when the specimen surface undergoes pure tensile loading is analyzed. The computed strain ratios for the aforementioned loading and boundary conditions are given in Table 2. It should be noted that these boundary conditions are different from those previously analyzed in the context of tensile loading.

**Figure 15:**
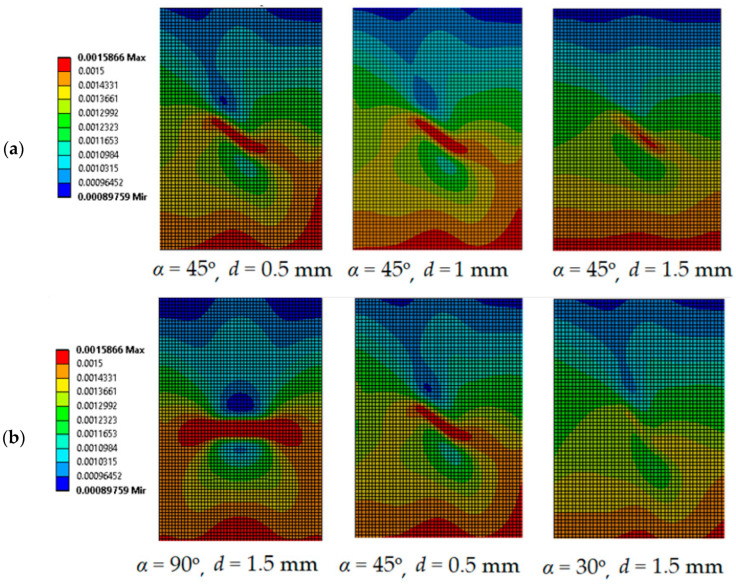
Longitudinal strain fields for different defect parameters at cantilever beam bending loading: (**a**)—various defect distances from surface; (**b**)—various defect angles.

From the results in Table 1 and Table 2, it can be observed that DIC measurements during cantilever beam bending can detect defects of length ≤ 6 mm twice as deep from the structure surface compared to tensile loading. However, only defects whose orientation angles are greater than 45° can be identified in the DIC strain field during cantilever beam bending loading. CFRP specimens with different defect longitudinal surface strain fields (*Y* direction, the same as in Figure 4) at bending loading are shown in Figure 15.

For cantilever beam bending loading, the defect distance from the surface *d* has no significant effect on the strain ratio *r*, as can be seen from the data in Table 2 and Figure 15a. The evaluation of the defect distance from the surface *d* from the bending strain field is difficult and, unlike tensile, cannot be accurately predicted. Furthermore, defects oriented perpendicular to the longitudinal direction of the specimen, show the best visibility (Figure 15b). Defects at *α* = 30° are not visible in the strain field. A linear relationship between strain ratio and defect length is also observed, as in the case of tensile loading.

## 4. Summary and Conclusions

This study presents a methodology based on Lamb waves and digital image correlation (DIC) for the detection and characterization of internal defects in composite structures. FEMU is an accurate method for defining defect geometry, as demonstrated by the determination of notch depth with an accuracy of 2%. Nevertheless, it should be noted that not all composite defects can be captured and characterized by DIC surface strain measurements. Therefore, additional testing, such as Lamb waves, may be required. The proposed Lamb waves—DIC methodology is based on the following:

1. The Lamb wave analysis must first be applied to the tested structure to identify defects in the structure. This can help to distinguish defects from noise in the DIC surface strain measurement. Once defects have been identified with Lamb waves and are visible in the surface strain field measured by DIC, DIC can be used to characterize defects and monitor their growth. The maximum to average strain ratio *r* around the defect is used as a defect characterization criterion in this study. A defect is visible if *r* is greater than 1.2 and its generated strain field unevenness is greater than the tested composite surface roughness.
2. The visibility of defects in the DIC strain field depends on the loading type of the structure, the defect location, the geometry, and the composite material. It has been demonstrated on the CFRP composite that tensile and cantilever beam bending loading have different defect visibility limits, e.g., tensile loading has an advantage over bending for defects that have a 1.5 times smaller angle to the loading direction, while bending has an advantage for the detection of deeper defects (75% of the structure thickness and more vs. a maximum limit of 65% of the structure thickness for tensile loading) from the structure surface. The minimum visible defect length is 10% of the structure width, but the unevenness of the composite surface limits the very minimum defect size to 3.8 mm (15% of the structure width) for the current CFRP.
3. Due to the variety of different composite materials, their lay-ups, and the infinite number of structural boundary conditions, this study proposes a methodology for the analysis of structure defects with DIC. A validated FEM of the structure can be used to create a strain field database (similar to Table 1 or Table 2), analyze defects of different geometries in a particular composite structure, and determine if such defects can be detected, characterized, monitored by DIC, or require other testing methods.

## Figures and Tables

**Table 1:** Strain ratios for different defect geometries during tensile loading.

	Angle	0				30				45				60				90			
	Length	4	5	6	10	4	5	6	10	4	5	6	10	4	5	6	10	4	5	6	10
Distance from surface	0.4	1.09	1.13	1.17	1.28	1.45	1.50	1.55	1.70	1.45	1.50	1.55	1.70	1.46	1.50	1.56	1.71	1.45	1.49	1.55	1.69
	0.5	1.00	1.00	1.01	1.10	1.26	1.29	1.34	1.47	1.26	1.29	1.34	1.47	1.26	1.30	1.35	1.47	1.25	1.29	1.34	1.46
	0.6	1.00	1.00	1.00	1.00	1.14	1.17	1.21	1.33	1.14	1.17	1.21	1.33	1.14	1.17	1.22	1.33	1.13	1.17	1.21	1.32
	0.8	1.00	1.00	1.00	1.00	1.10	1.13	1.17	1.28	1.10	1.13	1.17	1.28	1.10	1.13	1.18	1.29	1.09	1.13	1.17	1.28
	1	1.00	1.00	1.00	1.00	1.08	1.12	1.16	1.27	1.08	1.12	1.16	1.27	1.09	1.12	1.16	1.27	1.08	1.11	1.15	1.26
	1.2	1.00	1.00	1.00	1.00	1.07	1.10	1.14	1.25	1.07	1.10	1.14	1.25	1.07	1.10	1.14	1.25	1.06	1.09	1.13	1.24
	1.4	1.00	1.00	1.00	1.00	1.00	1.03	1.07	1.17	1.00	1.03	1.07	1.17	1.01	1.04	1.08	1.18	1.00	1.03	1.07	1.17
	1.5	1.00	1.00	1.00	1.00	1.00	1.00	1.03	1.13	1.00	1.00	1.03	1.13	1.00	1.00	1.04	1.14	0.97	1.00	1.03	1.13

					Not visible									Visible							


**Table 2:** Strain ratios for different defect geometries during bending loading.

	Angle	0				30				45				60				90			
	Length	4	5	6	10	4	5	6	10	4	5	6	10	4	5	6	10	4	5	6	10
Distance from surface	0.4	1.02	1.02	1.04	1.11	1.02	1.04	1.06	1.13	1.16	1.18	1.20	1.28	1.35	1.37	1.40	1.49	1.55	1.58	1.61	1.72
	0.5	1.00	1.00	1.02	1.08	1.00	1.01	1.03	1.10	1.13	1.15	1.17	1.25	1.31	1.34	1.36	1.46	1.51	1.54	1.57	1.68
	0.6	1.00	1.00	1.00	1.07	1.00	1.00	1.02	1.09	1.12	1.14	1.16	1.24	1.30	1.32	1.35	1.44	1.49	1.52	1.55	1.66
	0.8	1.00	1.00	1.00	1.07	1.00	1.00	1.02	1.09	1.11	1.13	1.16	1.23	1.29	1.32	1.34	1.44	1.49	1.51	1.55	1.65
	1	1.00	1.00	1.01	1.08	1.00	1.00	1.02	1.09	1.12	1.14	1.16	1.24	1.30	1.32	1.35	1.44	1.50	1.52	1.56	1.66
	1.2	1.00	1.00	1.00	1.07	1.00	1.00	1.02	1.09	1.11	1.13	1.16	1.24	1.30	1.32	1.35	1.44	1.49	1.52	1.55	1.66
	1.4	1.00	1.00	1.00	1.05	1.00	1.00	1.00	1.07	1.09	1.11	1.13	1.21	1.27	1.29	1.32	1.41	1.46	1.49	1.52	1.62
	1.5	1.00	1.00	1.00	1.03	1.00	1.00	1.00	1.04	1.07	1.09	1.11	1.19	1.24	1.26	1.29	1.38	1.43	1.45	1.48	1.59

					Not visible									Visible							


## Data Availability

Data are contained within the article.
